# Fresh Upper Gastrointestinal Bleeding (Hematemesis), a Rare Manifestation of Retrograde Intussusception Following Classic Gastric Bypass Surgery

**DOI:** 10.1002/ccr3.70511

**Published:** 2025-05-19

**Authors:** Nader Moeinvaziri, Naser Afshin, Nazanin Setayeshpour, Mohammad Ebrahimi

**Affiliations:** ^1^ Laparoscopy Research Center Shiraz University of Medical Sciences Shiraz Iran

**Keywords:** bariatric surgery, hematemesis, intussusception, Roux‐en‐Y gastric bypass

## Abstract

Retrograde intussusception may occur following Roux‐en‐Y gastric bypass surgery with a variety of presentations including colicky abdominal pain, constipation, and vomiting. A rare manifestation of this complication is upper gastrointestinal bleeding (hematemesis) which can cause misdiagnosis.

## 1 Introduction

Bariatric surgery (BS) is considered the most efficient long‐term therapy for morbid obesity [1]. Laparoscopic Roux‐en‐Y gastric bypass (RYGB) is generally considered a safe and effective procedure to prevent long‐term complications of morbid obesity [2]. Some complications such as bleeding, anastomosis stricture, marginal ulcers, leakage from the anastomosis site, and intussusception have been reported after gastric bypass [2, 3].

Intussusception is rare in adults, accounting for just 5% of all cases, and 90% of them, are due to an underlying pathology (like tumors or inflammatory processes, and polyps) acting as a leading point, causing invagination of the proximal part of the bowel into the distal alongside peristalsis (anterograde) [4]. Retrograde intussusception mostly follows Roux‐en‐Y gastric bypass [5]. In the RYGB, the distal bowel (common channel) is drawn into the lumen of the proximal bowel (alimentary limb or jejunojejunostomy anastomosis). These intussusceptions usually occur without a leading point and may represent a motility disorder following the Roux‐en‐Y reconstruction, which may require surgical intervention [6].

Diagnosis of intussusception is difficult due to its presentation with a wide range of symptoms and various severities, including acute or chronic abdominal pain and obstructive symptoms, and laboratory test results are not specific [7, 8]. In this study, we represent a 19‐year‐old woman with an unusual presentation of intussusception presenting as upper gastrointestinal bleeding (Hematemesis), which can cause misdiagnosis.

### 1.1 Case Presentation

A 19‐year‐old woman with a body mass index (BMI) of 47 (137 kg weight and 172 cm height) underwent RYGB surgery. The procedure was done by a standard method with 5 ports. A gastric pouch was created by three 60 mm Endo‐GI purple staplers, a gastrojejunal anastomosis was about 2 cm with an alimentary limb of 130 cm, and a biliopancreatic limb of 70 cm. Peterson and jejunojejunal defects were closed completely.

Diet was started for her the next day, and she was discharged the second day after surgery in a stable condition with instructions to have regular postoperation follow‐ups. She weighed 109 kg and 93 kg at 3 and 6 months postoperation follow‐ups, respectively. Iron deficiency anemia was detected in routine lab tests, and iron supplements were prescribed for her. Following her 6‐month follow‐up, she presented with low‐intensity colicky abdominal pain episodes after food ingestion that resolved spontaneously after a few minutes. However, she neglected the painful episodes as they were self‐limited.

**FIGURE 1:**
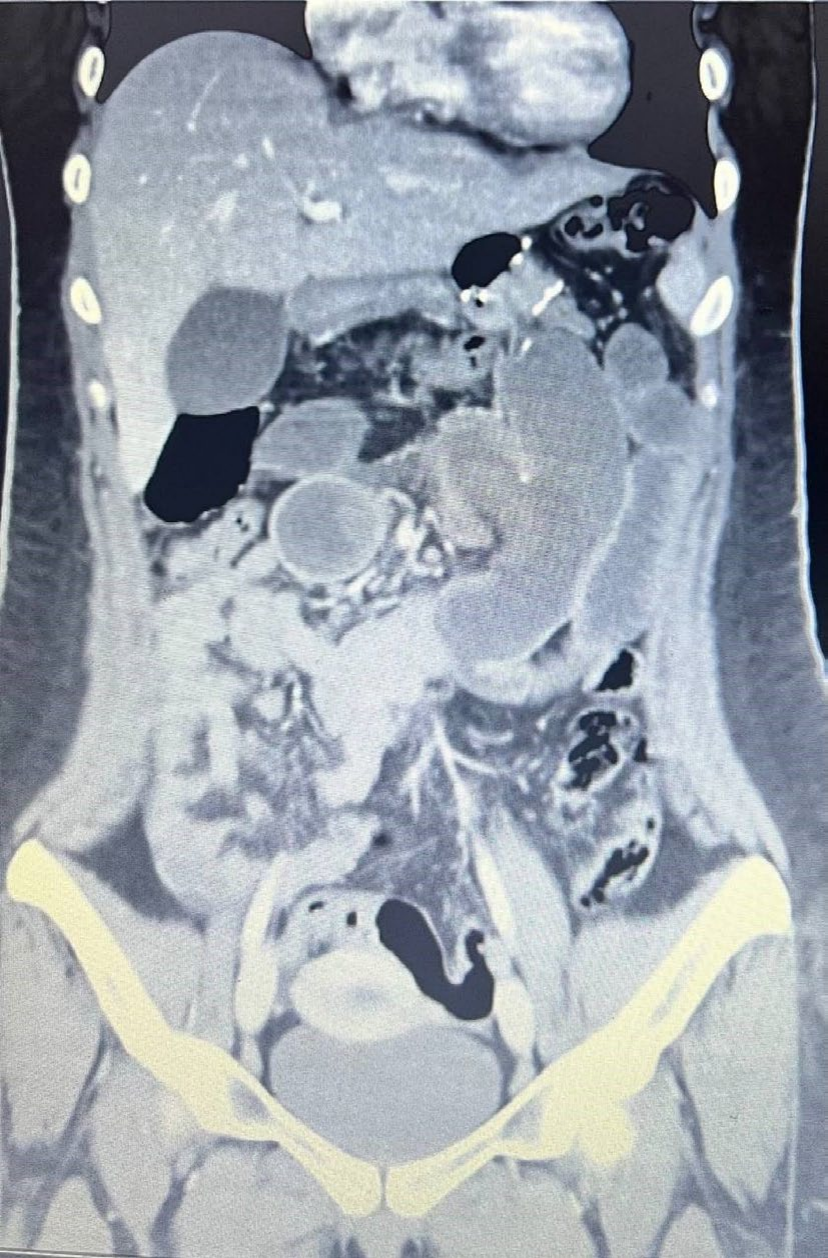
Abdominal CT demonstrating the target sign of a small‐bowel intussusception.

**FIGURE 2:**
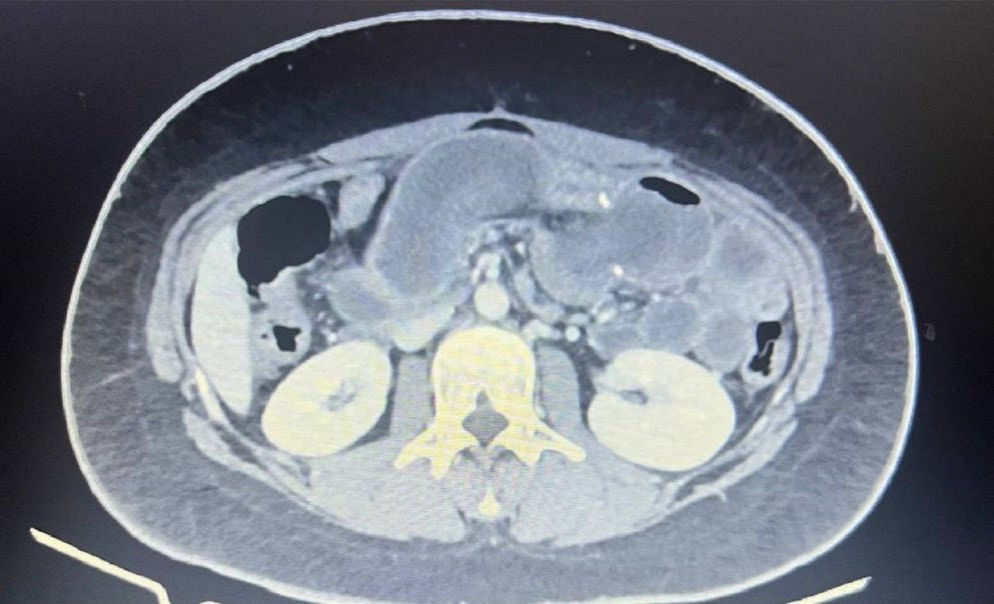
Axial abdominal CT demonstrating the classic target/bull's‐eye sign.

**FIGURE 3:**
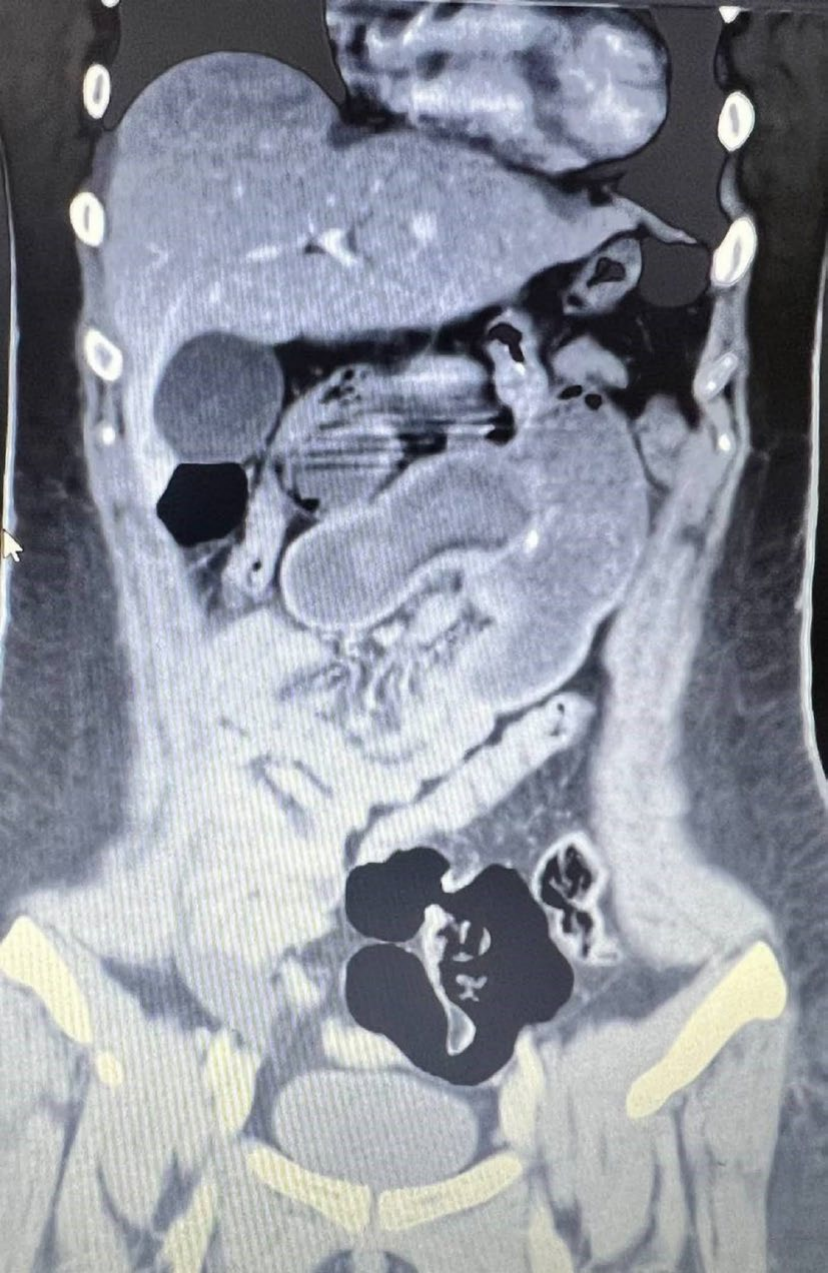
Coronal view of small bowel intussusception.

Nine months after the surgery, she was admitted to the hospital due to 2 episodes of massive bloody vomiting and abdominal pain (worse than her previous pains). Her vital signs were stable upon admission, and she had a low‐intensity colicky pain in the left upper quadrant without tenderness. She was scheduled for an upper gastrointestinal endoscopy, which showed a marginal ulcer at the site of the gastrojejunostomy anastomosis without active bleeding. The next day, her abdominal pain worsened along with upper abdominal tenderness during her physical examination. Her laboratory data showed elevated white blood cells from 8500 to 12,000 / μL and a mild decrease in hemoglobin concentration from 11 to 10.5 g/dL. An abdominal and pelvic computed tomography (CT scan) with contrast was done, which showed distention in small bowel lumen with a target sign (Figures 1, 2, 3).

With suspicion of intestinal obstruction and intussusception, the patient underwent laparotomy. Her surgical findings were retrograde intussusception of the common channel at the jejunojejunal anastomosis site with severe edema of the small intestine, which made bowel reduction impossible due to patchy areas of necrosis in the intestine. Consequently, the beginning of the common channel and the jejunojejunal anastomosis sites were resected, and Roux‐en‐Y configuration was re‐established. First, the end of the common channel anastomosed to the Roux limb, then the biliopancreatic limb connected to the common channel 30 cm distally, both in side‐toto‐side manner.

## 2 Outcome and Follow‐up

Her vital signs were stable 3 days after the surgery, and she tolerated a liquid diet after a normal dye study. On the fifth day after the operation, the drain was removed, and the patient was discharged in excellent condition. Two weeks after surgery, the patient visited without any definite complaints. she also visited after 3 months for her routine follow‐up, which had 6 kg weight loss and acceptable laboratory parameters.

## 3 Conclusion

In conclusion, a rare complication following Roux‐en‐Y gastric bypass surgery is retrograde intussusception with a variety of presentations including colicky abdominal pain, constipation, nausea, and vomiting, and even upper gastrointestinal bleeding (hematemesis) just like our case that can cause misdiagnosis during management. We believe that this paper is the first jejunojejunal intussusception presenting itself with hematemesis that is reported in the literature following RYGB surgery. Clinicians should suspect the presence of intussusception after RYGB even with fresh upper gastrointestinal bleeding presentation along with abdominal pain.

## 4 Discussion

Intussusception is rare in adults, and retrograde intussusception is even less common, with a variety of nonspecific symptoms including bowel obstruction, constipation, abdominal distention, and more commonly, abdominal pain, causing the diagnosis to be a challenging endeavor [7, 9, 10]. The usual symptoms that patients present with are epigastric pain with discrete intensity and duration, nausea, and vomiting [9]. In addition, rare cases present with a triad of abdominal pain, hematochezia, and an abdominal mass [4]. Post‐RYGB transient colicky abdominal pain should be considered important, as it may be an early presentation of intussusception or internal hernias. Our patient complained of recurrent limited abdominal pain, which, if presented sooner, could have been prevented by earlier intervention from progressing to complications and necrosis. Leading points such as tumors or inflammatory processes and polyps are known etiologies of intussusception in adults. however, some other causes such as significant weight loss, disruption of the duodenal pacemaker post transection of the jejunum, dysmotility after RYGB, and existence of the stapler line as a leading point may predispose the patients post RYGB to intussusception [11] Branch et al. reported a Roux‐en‐Y gastric bypass patient with jejunojejunostomy intussusception who also had hematemesis; though in that case, a concurrent bleeding marginal ulcer was found to be the source [12] There has been a reported case of hematemesis post invagination of the efferent limb into the gastrojejunostomy anastomosis in a patient with a previous history of billroth II surgery due to a complicated peptic ulcer [13] To the best of our knowledge, this is the first reported jejunojejunal intussusception presenting itself with upper GI bleeding. The patient had no complaints of hematemesis or melena during the entire 9 months after the operation until the time of hospitalization and only complained of colicky abdominal pain. During the endoscopy procedure, no evidence of active bleeding was detected at the anastomotic site. Also, during surgery and bowel resection, the secretions inside the intestine were bloody. We hypothesize that, due to the obstruction distal to the jejunojejunal anastomosis, the bloody secretions moved towards the alimentary limb and manifested as hematemesis. Given the above evidence, we assume that the bleeding is caused by vascular congestion, mucosal ischemia, and hemorrhage within the small intestine following intussusception. Although examination and laboratory data do not specify the diagnosis and ultrasound usually is not helpful in adult cases of intussusception, a CT scan with contrast can be the best modality for such cases, showing “target sign” which is suggestive of intussusception diagnosis [9, 14]. The pathology of the resected bowel also was in favor of transmural necrosis without any definite underlying cause.

The decision of surgical or conservational management of these cases depends on the patient's condition and the complications that follow. Some cases have shown a spontaneous reduction of intussusception; however, in other cases, bowel blockage, bowel ischemia, and bowel distention have been reported, causing necessary and emergency surgical management [9]. The challenging choice for the management of such cases can be eased with contrast abdominal CT scans [14]. Different surgical management choices have been suggested in the literature for intussusception, including intussusception reduction and resection of the damaged bowel with re‐establishing anastomosis [15]. Although some studies have reported enteropexy or mesenteric fixation to prevent the intussusception post RYGB [8], no definite recommended method for prevention has been reported to date. Ankur Makani et al. reported a case of recurrent intussusception post RYGB, which underscores the importance of understanding the causes for better diagnosis and prevention [11].

## Author Contributions

**Naser Afshin:** conceptualization, supervision, writing – original draft. **Nazanin Setayeshpour:** writing – review and editing. **Mohammad Ebrahimi:** data curation, resources. **Nader Moeinvaziri:** project administration, supervision, writing – original draft, writing – review and editing.

## Consent

Written informed consent was obtained from the patient and her family.

## Conflicts of Interest

The authors declare no conflicts of interest.

## Data Availability

The data that support the findings of this study are available on request from the corresponding author. The data are not publicly available due to privacy or ethical restrictions.
